# Physics-Guided Generative Inverse Design of Thermally Anisotropic Microstructures via FEM-Validated Conditional GANs

**DOI:** 10.21203/rs.3.rs-8703736/v1

**Published:** 2026-02-10

**Authors:** Yuhang Wu, Dongsheng Li, Rajendra K. Bordia, Fanchen Meng, Hai Xiao, Fei Peng

**Affiliations:** 1 D.W. Daniel High School, 140 Blue and Gold Blvd, Central, SC 29630, USA; 2 Advanced Manufacturing LLC, East Hartford, CT 06108, USA; 3 Department of Materials Science and Engineering, Clemson University, Clemson, SC 29634, USA; 4 Watt Family Innovation Center, Clemson University, Clemson, SC 29634, USA; 5 Department of Electrical and Computer Engineering, Clemson University, Clemson, SC 29634, USA; 6 Center for Optical Materials Science and Engineering Technologies (COMSET), Clemson University, Anderson, SC 29625, USA

**Keywords:** Inverse design, Thermal anisotropy, Microstructure design, Finite element method (FEM), Physics-guided machine learning

## Abstract

Inverse design problems governed by physical laws are challenging due to the nonlinear, high-dimensional, and non-unique relationships between the internal structure and the macroscopic response. While data-driven generative models have been applied to inverse microstructure design, maintaining physical consistency and scalability remains an important consideration for practical use.

In this work, we present a physics-guided generative inverse design framework that combines finite element method (FEM) simulations with a conditional Wasserstein generative adversarial network (GAN) to generate microstructural images conditioned on target physical properties. Rather than incorporating governing equations directly into the learning objective, physical laws are enforced through FEM-based data generation and assessed through closed-loop FEM re-simulation of the generated microstructures. All forward simulations are carried out using FEniCS, an open-source Python-based FEM platform that supports automated, scalable parallel execution.

The framework is demonstrated through the inverse design of thermally anisotropic microstructures containing parallel elliptical pores, a geometry motivated by its relevance to established ceramic processing routes. FEM validation indicates that the generated microstructures reproduce prescribed directional thermal conductivities with relative errors typically below 5–10% across a broad range of anisotropy conditions, while preserving key geometric characteristics without explicit geometric constraints.

These results suggest that physics-guided generative modeling provides a practical and flexible approach for inverse microstructure design in physically governed systems.

## 1. Introduction

Inverse problems governed by physical laws arise broadly in materials science and engineering, where internal microstructural states must be inferred or designed to achieve prescribed macroscopic behavior. Inverse design, the task of identifying material microstructures that yield target properties, has therefore become a central paradigm for accelerating materials discovery and optimization [1–3]. However, such problems are fundamentally challenging due to nonlinear, non-unique structure–property relationships and the high dimensionality of realistic microstructural representations.

Traditional inverse design approaches typically rely on manually defined microstructural descriptors, such as porosity, aspect ratio, or phase volume fraction, extracted from images or simulations [4–6]. While these descriptors provide compact statistical summaries, they inherently discard spatial and geometric information, limiting their ability to reconstruct complex microstructures. Higher-order features, including local anisotropy, connectivity, tortuosity, and spatial correlations, are difficult to encode using low-dimensional descriptors, even though they can have a decisive impact on effective material properties.

Recent advances in machine learning (ML) have enabled data-driven inverse generation frameworks that operate directly on high-dimensional representations, such as microstructural images, rather than predefined descriptors [7–9]. Among these, generative adversarial networks (GANs) have demonstrated strong capability in learning complex image distributions and generating realistic microstructures that preserve detailed geometric features, including shape, orientation, and connectivity [10–17]. Image-based generative models, therefore, provide a natural platform for inverse design, as they can represent diverse microstructures while accommodating the intrinsic non-uniqueness of inverse problems.

Building on this direction, the present authors have previously developed machine-learning- and GAN-based frameworks for predicting ceramic microstructures from indirect macroscopic or process-level signals, including hardness measurements and in situ optical monitoring during laser sintering [18–20]. These studies demonstrated that spatially meaningful microstructural information can be inferred from limited observables, motivating the extension of generative modeling toward physics-guided inverse generation, where microstructures are not only realistic but also physically consistent.

More broadly, physics-aware inverse design strategies have sought to incorporate governing equations into machine-learning workflows through surrogate property models, physics-regularized objectives, or guided generative sampling [17,21,22]. While these approaches can be effective for specific problem settings, they typically enforce physical constraints in an approximate manner and often require iterative optimization. In addition, their reliance on reduced-order representations or surrogate models can make it challenging to accommodate high-dimensional, spatially complex microstructures, particularly when rigorous validation using high-fidelity numerical solvers is required.

In contrast, the framework presented here adopts a physics-guided generative inverse paradigm in which governing equations are enforced directly through numerical simulation during both data generation and validation. Rather than embedding physical laws into the learning objective, the generative model is trained exclusively on physics-consistent data, and inverse predictions are verified through closed-loop finite-element-method (FEM) re-simulation. This strategy ensures strict physical consistency while retaining the efficiency and expressiveness of image-based generative modeling, enabling direct, one-shot inverse generation of high-resolution microstructures.

A critical requirement for such inverse generation is that the design space remains physically realistic and manufacturable. In this study, we therefore focus on microstructures composed of elliptical pores, which provide a compact yet expressive geometric representation. Elliptical pore morphologies naturally arise in several scalable ceramic processing routes, including directional freeze casting, extrusion-based processing, and templated sintering, in which pore elongation and orientation can be controlled while exact pore placement remains stochastic [23–25]. This makes elliptical pores well-suited for image-based generative modeling while maintaining consistency with realistic manufacturing constraints.

To demonstrate the proposed physics-guided inverse generation framework, we focus on the inverse design of thermally anisotropic microstructures, where effective thermal conductivity differs along orthogonal directions. Thermal anisotropy is of practical importance in applications such as thermal management, insulation, and energy systems, where directional heat transport is desirable [26–28]. By controlling the orientation, aspect ratio, and spatial distribution of elliptical pores, directional heat transport can be systematically tuned within a physically meaningful design space.

Using FEM simulations implemented in FEniCS (https://fenicsproject.org/), an open-source, Python-based finite-element framework, we compute effective thermal conductivities along orthogonal directions for microstructures containing parallel elliptical pores, generating a large, physics-consistent training dataset. The scriptable nature of FEniCS enables fully automated and independent simulation workflows that can be executed concurrently across multiple CPUs, facilitating scalable dataset construction for data-driven inverse design. Based on this dataset, a regression-based conditional Wasserstein GAN with gradient penalty (RCWGAN-GP) is trained to directly generate realistic microstructural images conditioned on target thermal conductivities. The inverse design accuracy is quantitatively assessed by re-simulating the GAN-generated microstructures using FEM, demonstrating physics-consistent, one-shot inverse design of strongly anisotropic materials.

The main contributions of this work are:

- a physics-guided inverse generative framework that enforces governing equations through closed-loop FEM validation rather than physics-informed losses;
- a scalable FEM-based dataset construction strategy with explicit bias mitigation for anisotropic inverse design;
- a comprehensive physical and geometric validation of inverse-generated microstructures, including conductivity accuracy, pore count, ellipticity, and orientation consistency.

## 2. Results and discussion

### 2.1 FEM Simulation Results and Training Data Distribution

The training dataset used for the GAN was generated entirely via finite element method (FEM) simulations of synthetically constructed microstructures. For each pore-count case (1, 2, 4, and 8 pores), 10,000 microstructures were generated and simulated to compute their effective thermal conductivities in the *x*- and *y*-directions. The example microstructures are given in Table 1. While this procedure yields a large, physics-consistent dataset, the resulting conductivity distribution is strongly influenced by the microstructure-generation process.

In the original random placement algorithm, smaller pores are significantly easier to place within a fixed 128 × 128 domain without intersecting neighboring pores or the domain boundaries. As a result, microstructures containing relatively small pores are generated with much higher probability than those containing large pores. Since small pores preserve solid connectivity in both directions, these microstructures tend to exhibit high effective thermal conductivities along both the *x*- and *y*-directions.

**Figure 1.**
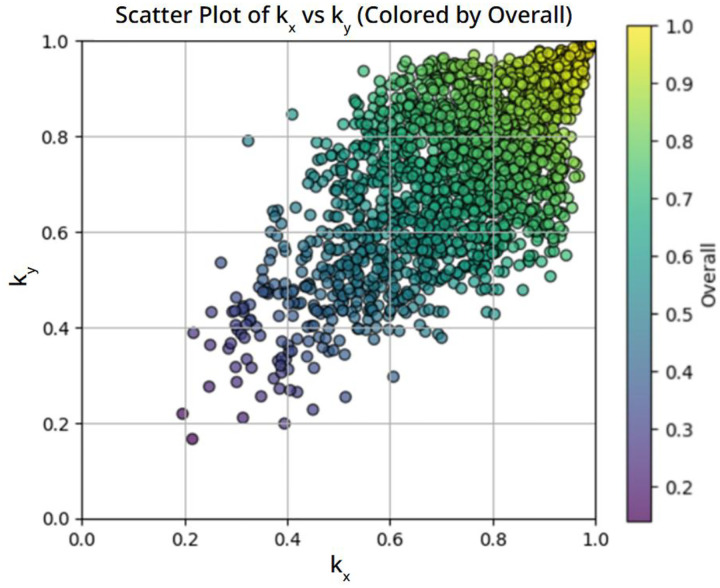
Joint distribution of FEM-computed thermal conductivities k_x_ and k_y_ for the original dataset, showing strong clustering near the isotropic line k_x_ ≈ k_y_. All conductivities are normalized by the matrix thermal conductivity

This bias is clearly illustrated in Figure 1, which shows the joint distribution of *k*_*x*_ and *k*_*y*_ for the original dataset. The majority of data points cluster tightly around the diagonal line *k*_*x*_ ≈ *k*_*y*_, indicating that most generated microstructures exhibit nearly isotropic thermal behavior. This strong bias toward isotropic properties is undesirable for the present study, as the primary objective is to investigate and, by inverse design, to create microstructures with pronounced thermal anisotropy.

As an initial attempt to increase the representation of anisotropic data, a simple data augmentation strategy was applied. Each microstructure in the original dataset was rotated by 90 degrees, and the rotated structure was added to the dataset. Because a 90-degree rotation exchanges the *x*- and *y*-directions, this operation effectively swaps the values of *k*_*x*_and *k*_*y*_while preserving the underlying geometry and physical consistency of the microstructure. This augmentation increases the dataset size without introducing artificial features.

**Figure 2.**
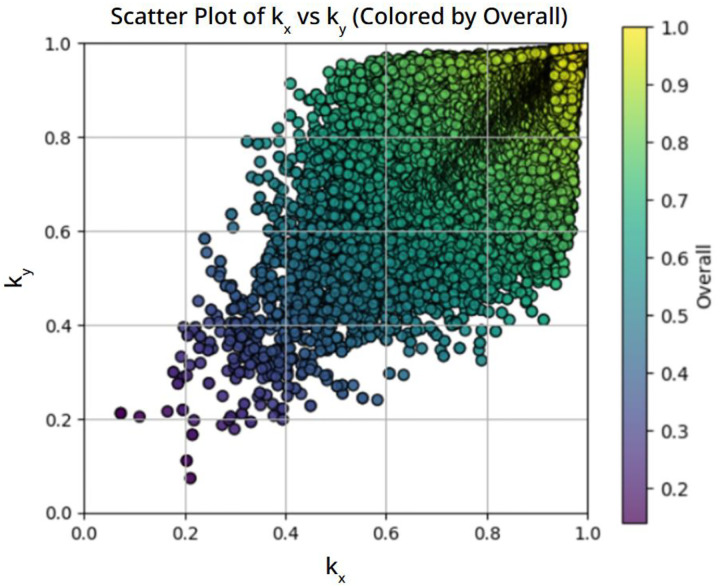
Conductivity distribution after applying 90-degree rotation–based data augmentation, which exchanges k_x_ and k_y_ while preserving microstructure geometry and physical consistency.

The effect of this rotation-based data augmentation is shown in Figure 2. Compared to the original distribution, the augmented dataset contains additional samples mirrored across the diagonal. While this procedure increases directional diversity, the overall distribution remains strongly concentrated near the isotropic line. Therefore, although rotation-based augmentation partially improves coverage of anisotropic conductivity pairs, it does not fundamentally eliminate the bias introduced by the original microstructure generation process.

To more effectively address this limitation, the microstructure generation algorithm itself was modified. Instead of selecting ellipse centers purely at random, ellipses were first compacted as tightly as possible into a grid-like configuration. Random center positions were then selected from this compacted arrangement, and small random perturbations were applied to introduce geometric variability. In addition, the semi-major and semi-minor axes of the ellipses were manually varied to encourage the placement of larger pores. These changes substantially increase the likelihood of generating microstructures with larger pores, which more strongly impede heat transport in one direction and thus promote thermal anisotropy.

**Figure 3.**
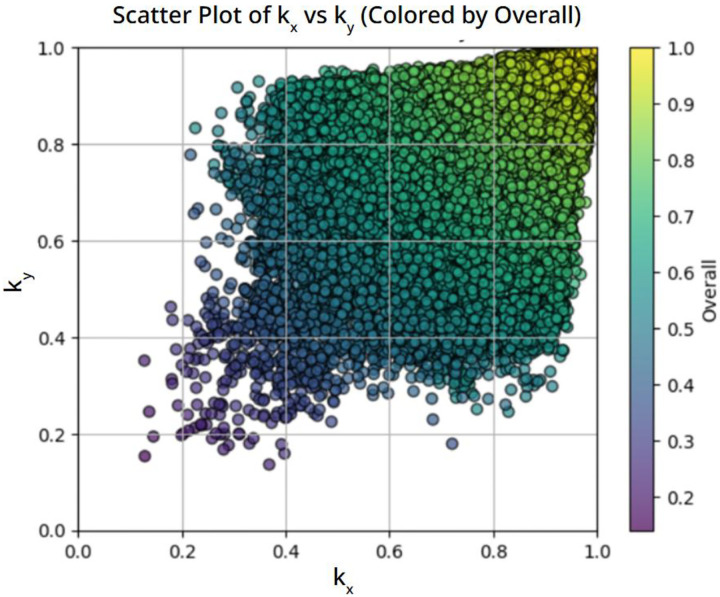
Conductivity distribution obtained after modifying the microstructure generation algorithm to favor larger pores, thereby increasing the representation of anisotropic thermal properties.

The conductivity distribution resulting from this modified generation strategy is shown in Figure 3. Compared to Figures 1 and 2, the dataset now spans a much broader region of the *k*_*x*_–*k*_*y*_ space. Strongly anisotropic microstructures, in which one directional conductivity is significantly lower than the other, are now well represented. This confirms that modifying the microstructure generation procedure is essential for producing training data suitable for anisotropic inverse design.

**Figure 4.**
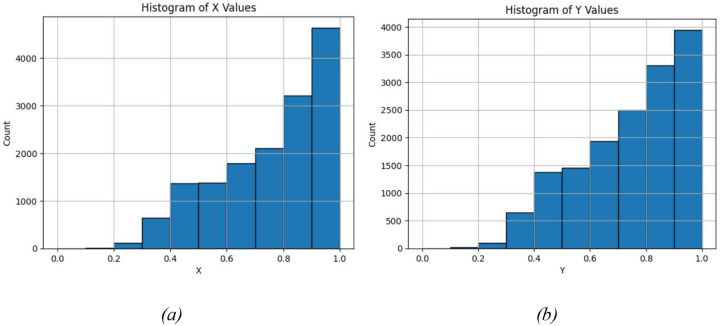
Marginal conductivity histograms illustrate residual overrepresentation of high-conductivity samples despite modified microstructure generation.

Despite this improvement, the dataset remains unevenly distributed. As shown in Figure 4, microstructures with higher thermal conductivity remain overrepresented. In particular, more than 4,000 samples exhibit *k*_*x*_ values between 0.9 and 1.0, whereas only approximately 1,500 samples fall within the range 0.5 < *k*_*x*_ < 0.6. This imbalance reflects the geometric constraints of the problem: achieving very low thermal conductivity requires extremely large pores, which are increasingly difficult to place without overlapping even under the modified generation scheme.

Such an uneven distribution can adversely affect GAN training by causing the model to overlearn features associated with high-conductivity microstructures while underperforming in sparsely sampled regions of the conductivity space. To mitigate this issue during training, an inverse bin sampling strategy was employed. The conductivity space was discretized into bins based on the values of *k*_*x*_ and *k*_*y*_, and training samples were selected with a probability inversely proportional to the number of samples in their corresponding bin.

**Figure 5.**
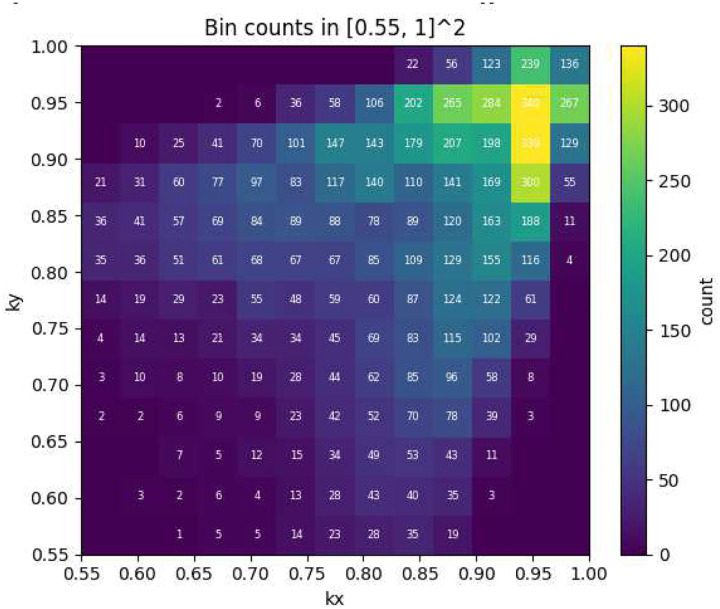
Effective training sample distribution after applying inverse bin sampling, demonstrating more uniform coverage of the conductivity space.

The effect of inverse bin sampling on the effective training distribution is illustrated in Figure 5, which shows the number of training samples contained in each bin of the twodimensional (*k*_*x*_, *k*_*y*_) conductivity space. Rather than modifying the underlying dataset, inverse bin sampling alters how frequently samples from different regions of the conductivity space are presented to the GAN during training. As shown in Figure 5, bins corresponding to sparsely populated regions, particularly those associated with strongly anisotropic thermal behavior, are assigned higher sampling weights, while bins with a large number of samples, typically associated with high and nearly isotropic conductivities, are sampled less frequently.

This bin-based sampling strategy results in a more balanced, effective training distribution across the conductivity domain 0.4 ≤ *k*_*x*_, *k*_*y*_ ≤ 1.0, despite the intrinsic imbalance of the raw dataset. By ensuring that microstructures from low-density bins are encountered more often during training, the GAN is prevented from overemphasizing high-conductivity, near-isotropic cases. Consequently, the model is evenly exposed to microstructures spanning both isotropic and strongly anisotropic thermal behaviors, which is essential for learning a robust, generalizable inverse mapping between thermal conductivities and microstructural geometry.

### 2.2 Overview of Generated Microstructures

After training, the conditional GAN was used to generate microstructures corresponding to prescribed target thermal conductivities. Representative examples are shown in Table 2 for cases exhibiting strong anisotropy in the x-direction, strong anisotropy in the y-direction, and near-isotropic thermal behavior. In all cases, the generated images show clearly defined pores embedded in a continuous solid matrix, consistent with the assumptions used in the FEM simulations.

Qualitative inspection indicates that the generated microstructures closely resemble those in the training dataset. The pores are approximately elliptical, exhibit comparable sizes within a given microstructure, and are generally aligned along a common orientation. These characteristics suggest that the GAN has learned the statistical and geometric features of the underlying microstructure distribution rather than reproducing individual training samples. Minor geometric deviations, such as slight variations in pore shape or spacing, are observed in a small fraction of cases, particularly for microstructures with higher pore counts. Such variations are expected and do not necessarily indicate reduced inverse design quality, as the relationship between microstructure geometry and effective thermal conductivity is inherently non-unique.

Because multiple distinct microstructures can yield similar thermal responses, visual similarity alone is insufficient to assess inverse design accuracy. Accordingly, all generated microstructures were re-simulated using the same FEM framework employed during dataset generation. This physics-based validation provides a direct and application-relevant measure of whether the generated microstructures satisfy the prescribed thermal conductivities, independent of superficial visual differences.

In addition to physical validation, the geometric validity and manufacturability of the generated microstructures were examined. For the class of materials considered, physically meaningful microstructures are expected to exhibit non-overlapping, approximately elliptical pores of comparable size, aligned along a common direction, and fully contained within the computational domain. Importantly, none of these geometric constraints were explicitly enforced during GAN training. The emergence of such features, therefore, indicates that the model has learned meaningful correlations between microstructural geometry and thermal properties from data alone.

As illustrated by the representative examples in Table 2, the generated microstructures consistently satisfy these geometric criteria across both isotropic and anisotropic thermal regimes. These qualitative observations provide a foundation for the quantitative validation of inverse design performance presented in the following sections.

### 2.3 FEM Validation of GAN-Generated Microstructures

This FEM re-simulation step distinguishes the proposed framework from surrogate-based inverse design approaches, as it provides a direct, physics-exact validation of generated microstructures rather than relying on learned property predictors.

While qualitative inspection confirms that the GAN-generated microstructures are geometrically realistic, a rigorous evaluation of inverse design performance requires quantitative validation based on physical properties. Because the relationship between microstructure geometry and effective thermal conductivity is non-unique, visual similarity alone is insufficient to assess whether the generated microstructures successfully realize the prescribed thermal behavior. Therefore, all GAN-generated microstructures were re-evaluated using finite element method (FEM) simulations to directly compute their effective thermal conductivities.

**Figure 6.**
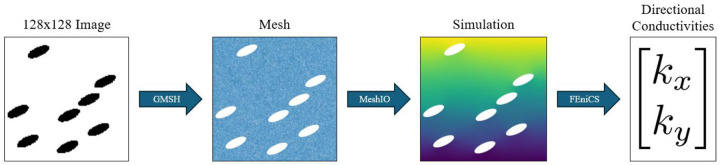
Validation process to re-simulate directional conductivities from a GAN-generated microstructure. The GMSH library converts the image into a mesh, which is then used for a FEM simulation. From this, FEniCS calculates the directional conductivities.

For each target conductivity pair (*k*_*x*_, *k*_*y*_), the GAN was used to generate a corresponding microstructure image. These images were then converted into FEM input geometries using the same preprocessing pipeline employed during dataset generation. The effective thermal conductivities in the *x*- and *y*-directions were computed using identical governing equations, boundary conditions, and numerical settings. This ensures that any discrepancy between target and predicted conductivities arises solely from the inverse design process, rather than from the inconsistencies in the physical model or numerical implementation. The re-simulation process for validating the GAN-generated microstructure is given in Figure 6.

**Figure 7.**
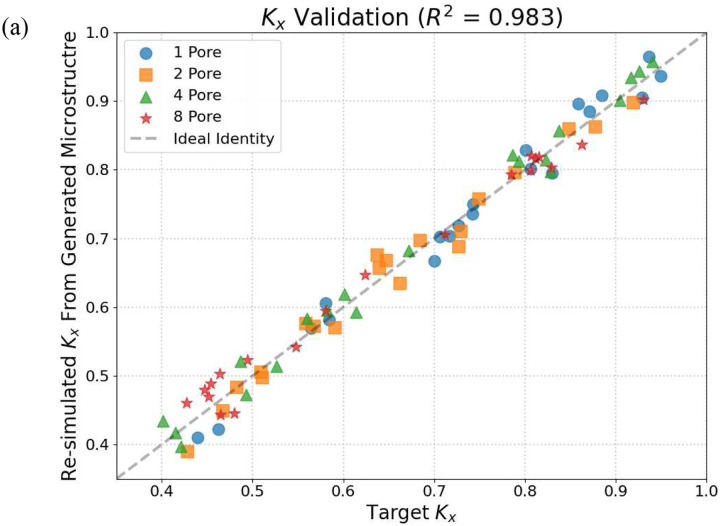

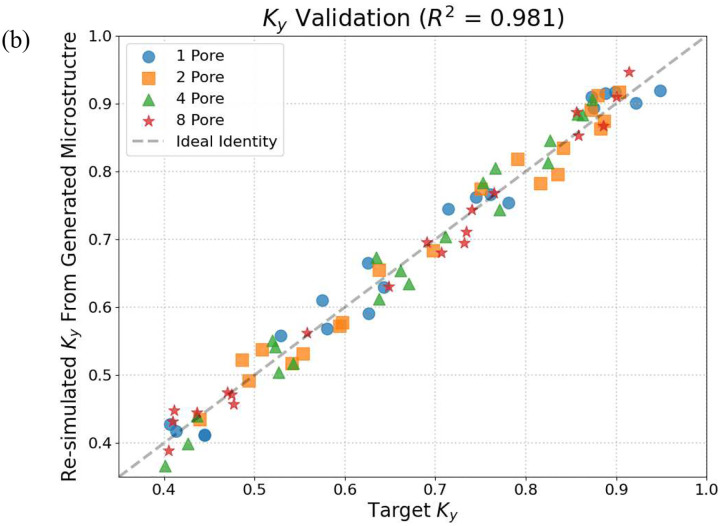
Comparison between target thermal conductivities and re-simulated conductivities of GAN-generated microstructures for both k_x_ and k_y_. The diagonal line indicates perfect agreement.

The comparison between target conductivities and FEM-computed conductivities of the GAN-generated microstructures is shown in Table 3 and Figure 7. Each data point corresponds to a single generated microstructure, with the horizontal axis representing the target conductivity and the vertical axis representing the FEM-validated conductivity. Results are shown for both *k*_*x*_ and *k*_*y*_, allowing directional accuracy to be assessed independently.

As shown in Figure 7, the FEM-computed conductivities closely track the target values across a wide range of conductivity pairs. Most data points lie near the diagonal line corresponding to perfect agreement, indicating that the GAN successfully generates microstructures whose effective thermal properties match the prescribed targets. For the majority of cases, the relative error in both *k*_*x*_ and *k*_*y*_ is below 5%, demonstrating high inverse design accuracy.

### 2.4 Effect of Pore Count on Inverse Design Accuracy

To further quantify performance, prediction errors were analyzed across different pore-count models (1, 2, 4, and 8 pores) and different anisotropy regimes. A summary of the median error and interquartile range (IQR) for each case is provided in Table 4. Three categories of target conductivities were considered: *x*-dominant anisotropy (*k*_*x*_ ≫ *k*_*y*_), near-isotropic behavior (*k*_*x*_ ≈ *k*_*y*_), and y-dominant anisotropy (*k*_*y*_ ≫ *k*_*x*_).

Across all pore counts and anisotropy regimes, the median errors for both *k*_*x*_ and *k*_*y*_ remain on the order of 10^−2^, indicating comparable inverse design accuracy. No monotonic dependence of median error on pore count is observed: while certain pore configurations yield lower errors for specific conductivity components or anisotropy regimes, no single pore count consistently minimizes the error across all cases.

Differences between models are more evident in the variability of prediction errors. Microstructures with very few pores exhibit greater sensitivity to small geometric perturbations, leading to higher error variability in some regimes, whereas microstructures with higher pore counts show greater dispersion in certain anisotropic cases, likely due to greater geometric complexity and closer pore spacing. Models trained on intermediate pore counts tend to exhibit more moderate variability.

Inverse design performance remains comparable for x-dominant, y-dominant, and near-isotropic targets, with no systematic loss of accuracy for strongly anisotropic cases. Overall, the results indicate that pore count primarily influences the spread of prediction errors rather than the median accuracy of inverse design.

### 2.5 Accuracy of Pore Count Reproduction

In addition to reproducing target thermal conductivities and generating geometrically valid pore shapes, a critical requirement for inverse microstructure design is that the generated microstructures respect the pore-count constraints imposed by the training data. In this study, separate GAN models were trained for microstructures containing 1, 2, 4, and 8 pores. Ideally, each model should generate microstructures with the same number of pores as those present in its corresponding training dataset.

To quantitatively evaluate pore-count accuracy, an automated image analysis procedure was applied to the GAN-generated microstructures. A computer vision-based algorithm was used to identify and count distinct pores within each image. Only pores that were fully enclosed within the domain, did not intersect neighboring pores, and exceeded a minimum size threshold were included in the count. This filtering step ensures that the pore-count analysis reflects physically meaningful pores rather than spurious artifacts.

**Figure 8.**
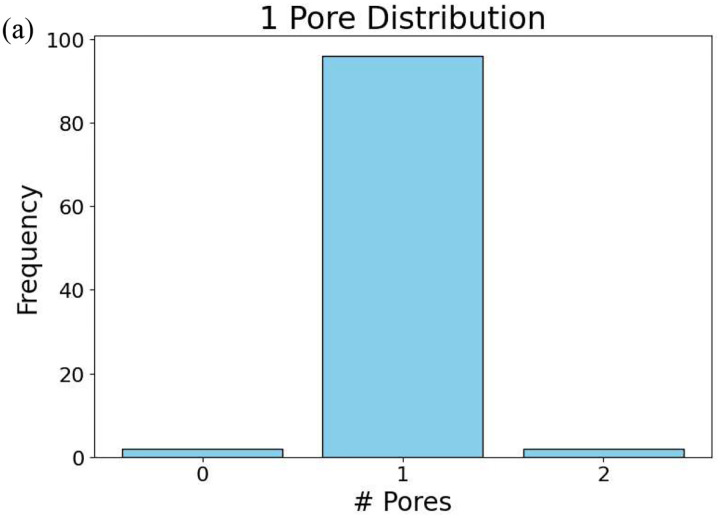

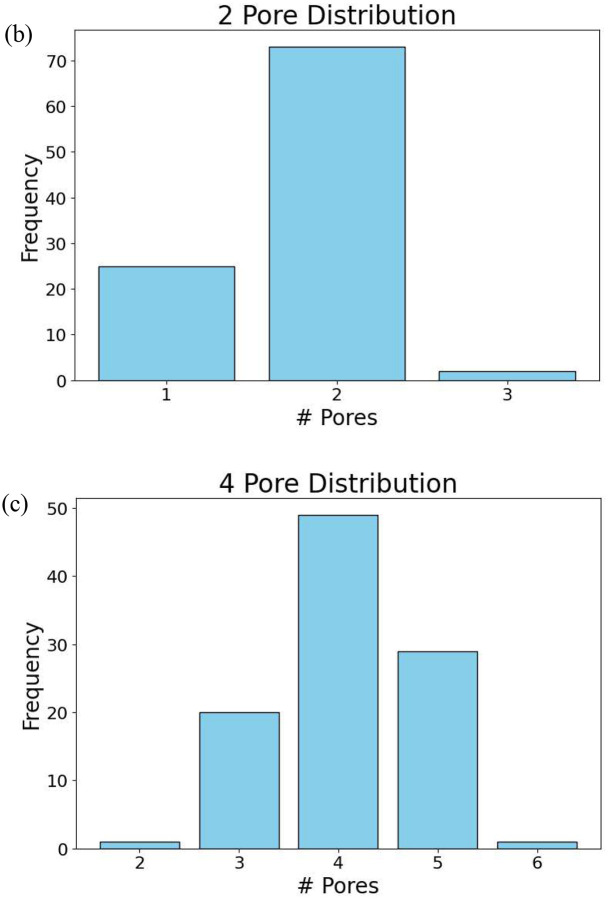

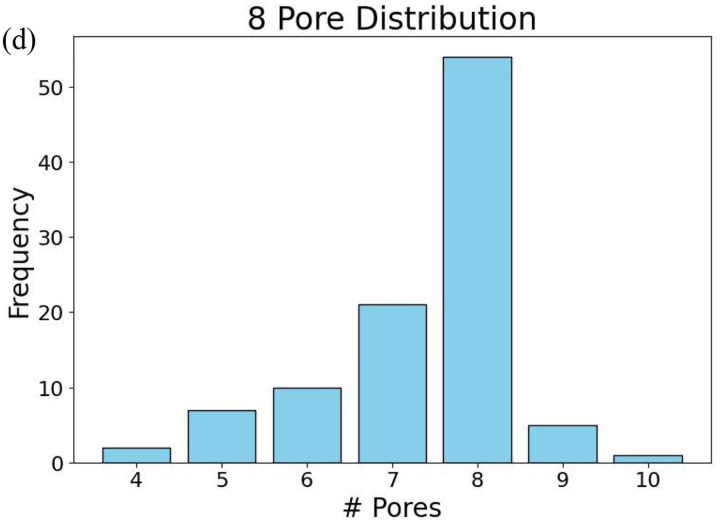
Distribution of detected pore counts for GAN-generated microstructures trained on datasets containing 1, 2, 4, and 8 pores. For each model, 100 generated microstructures were analyzed using an automated pore-detection algorithm.

For each GAN model, 100 microstructures were randomly generated using randomly selected target conductivity pairs. The number of pores detected in each generated microstructure was then recorded and compared with the expected pore count in the training dataset. The resulting distributions of detected pore counts are shown in Figure 8.

As shown in Figure 8, the most frequently detected pore count for each GAN model corresponds to the pore number used during training. The single-pore model exhibits the highest accuracy, with approximately 95% of the generated microstructures containing exactly one detected pore. This high accuracy reflects the relatively simple geometry of the one-pore case, where the pore is large, well separated, and easily identified by the detection algorithm.

As the number of pores increases, the accuracy of pore-count reproduction gradually decreases. For the two-pore and four-pore models, the majority of generated microstructures still contain the correct number of pores, although a small fraction of images exhibit one fewer or one additional detected pore. These deviations are typically observed when pores are generated very close to one another, leading to partial merging or ambiguous boundaries during image processing.

The eight-pore model shows the largest deviation from the ideal pore count, with approximately 60% of generated microstructures containing exactly eight detected pores. This reduction in accuracy is primarily due to the smaller size and closer spacing of individual pores in the eight-pore configuration. Under these conditions, small geometric variations can cause neighboring pores to appear connected or cause very small pores to fall below the detection threshold.

Importantly, deviations in the detected pore count do not necessarily imply a failure of the inverse design process. Rather, they reflect a combination of increased geometric complexity at higher pore counts and the limitations of automated pore-detection algorithms when applied to small, closely spaced features. Visual inspection confirms that, even when the detected pore count differs from the nominal training value, the generated microstructures remain physically reasonable and consistent with the overall geometric characteristics of the training data.

More importantly, FEM-based validation demonstrates that these geometric deviations do not compromise the physical accuracy of the generated designs. As shown in Table 4, inverse design errors in the effective thermal conductivities remain comparable across pore counts, even when the detected pore number deviates from the training value. This indicates that the governing heat transport behavior is preserved even when discrete geometric features, such as pore count, are not reproduced exactly.

These results highlight an important aspect of physics-guided inverse design: accurately reproducing target physical properties does not require the exact reconstruction of every geometric detail. Instead, the conditional GAN learns to generate microstructures that satisfy the prescribed thermal response, consistent with the inherent non-uniqueness of the inverse problem. The ability to maintain physical fidelity despite variations in discrete geometric features further supports the robustness of the proposed framework.

### 2.6 Ellipticity of Generated Pores

Beyond reproducing the correct number of pores, an additional requirement for physically realistic and manufacturable microstructures is that the generated pores closely resemble the intended elliptical geometry. In the microstructures considered in this study, pore ellipticity is particularly important because effective thermal conductivity is sensitive not only to pore size and orientation, but also to deviations from idealized pore shape. Moreover, many fabrication techniques that produce aligned porous structures naturally yield pores that are approximately elliptical rather than irregular.

To quantitatively assess the shape fidelity of the GAN-generated pores, an ellipticity metric was introduced. For each detected pore, a best-fit ellipse was computed using image-processing techniques. The ellipticity of an individual pore was defined as the fraction of the pore area that lies within the fitted ellipse, resulting in a normalized value between 0 and 1. A value of 1 corresponds to a perfect ellipse, while lower values indicate increasing deviation from ideal elliptical geometry. The ellipticity of a generated microstructure was computed as the average ellipticity of all detected pores within the image.

**Figure 9.**
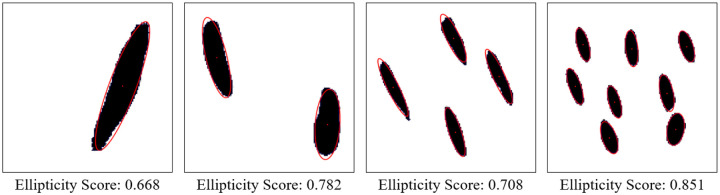
Representative examples of ellipticity evaluation for GAN-generated microstructures with one, two, four, and eight pores. Best-fit ellipses are overlaid on detected pore boundaries, illustrating close agreement between generated pore shapes and ideal elliptical geometry.

Representative examples of the ellipticity evaluation procedure are shown in Figure 9, which presents generated microstructures with different numbers of pores. In these examples, the best-fit ellipses are overlaid on the detected pore boundaries. Visual inspection shows close agreement between the generated pore shapes and their fitted ellipses, indicating that the pores produced by the GAN are smooth and well approximated by elliptical geometries, even for cases with a larger number of pores.

**Figure 10.**
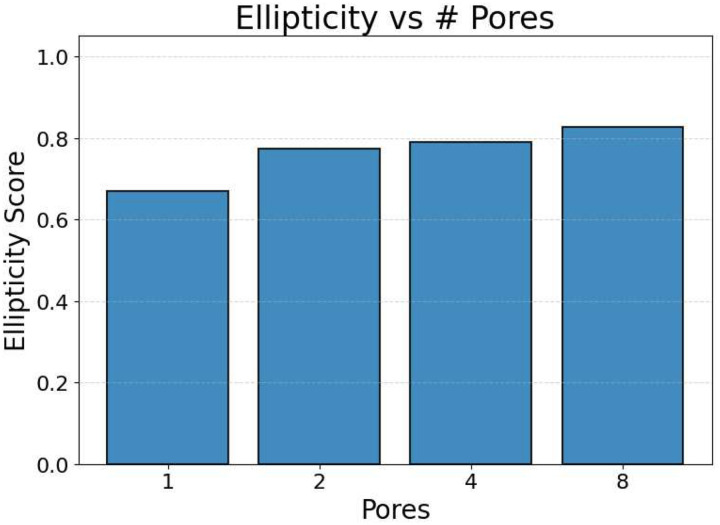
Statistical distribution of average pore ellipticity for GAN-generated microstructures. Results are shown for 100 randomly generated samples for each pore-count model, demonstrating consistently high ellipticity across all cases.

To assess pore shape fidelity statistically, ellipticity analysis was performed on 100 randomly generated microstructures for each GAN model. The resulting distributions of average pore ellipticity are summarized in Figure 10. Across all pore-count cases, the generated microstructures exhibit consistently high ellipticity values, indicating that the GAN preserves the intended pore shape. The average ellipticity is approximately 0.8 for single-pore microstructures and increases to approximately 0.9 for microstructures containing eight pores.

Rather than indicating a fundamental change in pore geometry, this difference reflects the manner in which shape information is reinforced during training. In microstructures with a larger number of pores, elliptical shape characteristics are repeatedly represented within a single training sample. This repeated exposure effectively reinforces elliptical geometry during learning, making deviations from the target shape less likely in the generated outputs. In contrast, single-pore microstructures provide only one instance of the target shape per sample, making the measured ellipticity more sensitive to local geometric variations along a larger pore boundary.

Importantly, pore ellipticity was not explicitly encoded in the GAN loss function or enforced through post-processing. The consistently high ellipticity values observed across all pore-count cases, therefore, indicate that the GAN has implicitly learned the association between elliptical pore geometry and the corresponding thermal response from the physics-consistent training data. This observation is consistent with the qualitative examples shown in Figure 9 and supports the conclusion that the model captures meaningful structure–property relationships rather than relying on superficial visual cues.

Overall, the ellipticity analysis demonstrates that the proposed inverse design framework can generate microstructures with reliable pore-shape fidelity across varying geometric complexities. Together with the pore-count and orientation analyses, these results confirm that the GAN-generated microstructures satisfy key geometric and manufacturability considerations while achieving the prescribed thermal properties.

### 2.7 Orientation Consistency of Generated Pores

In addition to pore count and pore shape, the relative orientation of pores within a microstructure is a critical geometric characteristic for both physical performance and manufacturability. For the class of materials considered in this study, the pores are intended to be approximately parallel, reflecting the assumptions used in data generation and the constraints imposed by common fabrication processes capable of producing directionally aligned porosity. Misalignment of pores within a single microstructure can significantly alter heat-flow pathways and degrade the intended anisotropic thermal response.

To evaluate whether the GAN preserves this alignment behavior, the orientation of each pore was extracted using the same best-fit ellipse procedure employed in the ellipticity analysis. For each detected pore, the orientation angle was defined as the angle between the major axis of the fitted ellipse and the horizontal direction. This procedure yields a set of orientation angles for each generated microstructure.

**Figure 11.**
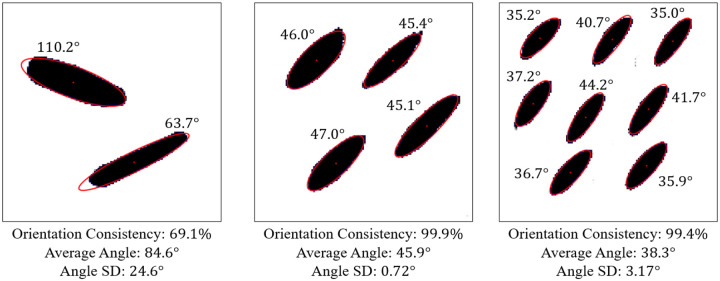
Representative GAN-generated microstructures illustrating pore orientation consistency. Best-fit ellipses and their major-axis orientations are shown, demonstrating alignment of pores within each microstructure.

Representative examples illustrating pore orientation consistency are shown in Figure 11, which presents several GAN-generated microstructures along with the corresponding fitted ellipse orientations. Visual inspection shows that, within each microstructure, the pores are largely aligned along a common direction. This alignment is observed consistently across microstructures generated for different target thermal conductivities and different pore counts.

To quantitatively assess orientation consistency, statistical measures were computed for each generated microstructure. Specifically, the mean orientation angle and standard deviation of the pore orientations were calculated. An orientation consistency metric was then defined as the fraction of pores whose orientation deviates from the mean by less than a prescribed angular tolerance. This metric provides a normalized measure of how well the pores within a microstructure are aligned.

**Figure 12.**
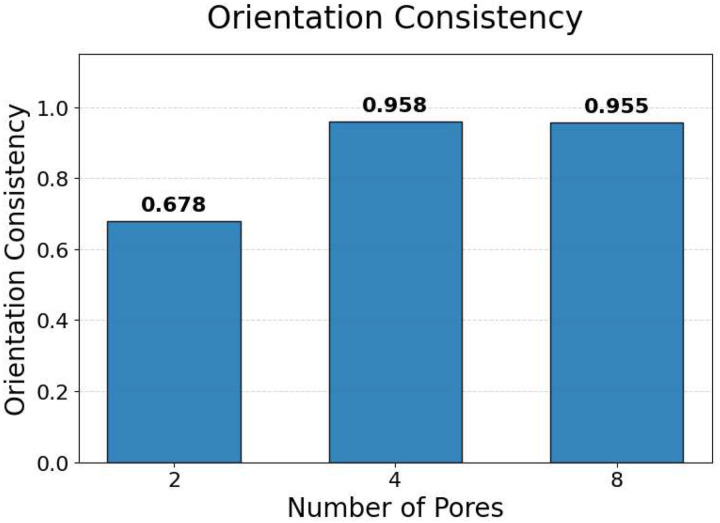
Statistical orientation consistency of GAN-generated microstructures. Results are shown for 100 randomly generated samples for each pore-count model (excluding the single-pore case), indicating consistently high pore alignment across all configurations.

The statistical results of this analysis are summarized in Figure 12, which shows the orientation consistency computed over 100 randomly generated microstructures for each GAN model (excluding the single-pore case, for which orientation consistency is not meaningful). Across all pore-count cases, orientation consistency remains high, typically above 90% for four and eight-pore microstructures. However, the 2-pore microstructure has much less consistency (67.8%). This is probably due to the large number of pores exposed to the model during training with 4- and 8-pore microstructures. When the model is exposed to fewer pores, it performs worse at generating perfect ellipses and alignments.

The orientation analysis provides complementary insight into how the generative model encodes directional information relevant to anisotropic heat transport. As shown in the qualitative examples (Figure 11) and the statistical distributions (Figure 12), the generated microstructures exhibit a high degree of orientation consistency within each sample, despite the absence of any explicit alignment constraint during training.

Rather than reflecting direct geometric supervision, this behavior arises from the strong coupling between pore orientation and directional thermal conductivity in the underlying physics. To achieve the prescribed anisotropic thermal response, the generator must produce microstructures with coherent directional features, of which pore alignment is a dominant contributor. The observed orientation consistency, therefore, indicates that the GAN has learned to encode physically meaningful directional patterns that support the target heat-transport behavior.

When considered alongside the pore-count and ellipticity analyses, the orientation results demonstrate that the proposed framework captures multiple, interrelated geometric characteristics required for physically realistic, manufacturable microstructures. Importantly, these characteristics emerge naturally from physics-consistent training data rather than from explicit geometric constraints. This reinforces the conclusion that the inverse design framework prioritizes physical fidelity while allowing geometric details to adapt flexibly to satisfy the prescribed thermal properties.

### 2.8 Training Dynamics and Model Stability

The training dynamics of the conditional GAN provide important insight into both model stability and convergence behavior. In this study, each GAN model (corresponding to 1, 2, 4, and 8 pores) was trained for 1,000 epochs using the same architecture and training protocol. To evaluate training progress in a physically meaningful way, the model performance was monitored by periodically generating microstructures from randomly selected target conductivity pairs and re-evaluating their effective thermal conductivities using FEM simulations.

**Figure 13.**
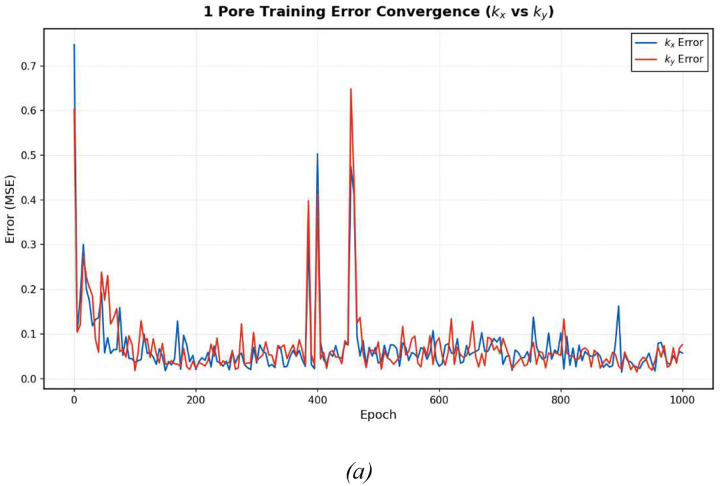

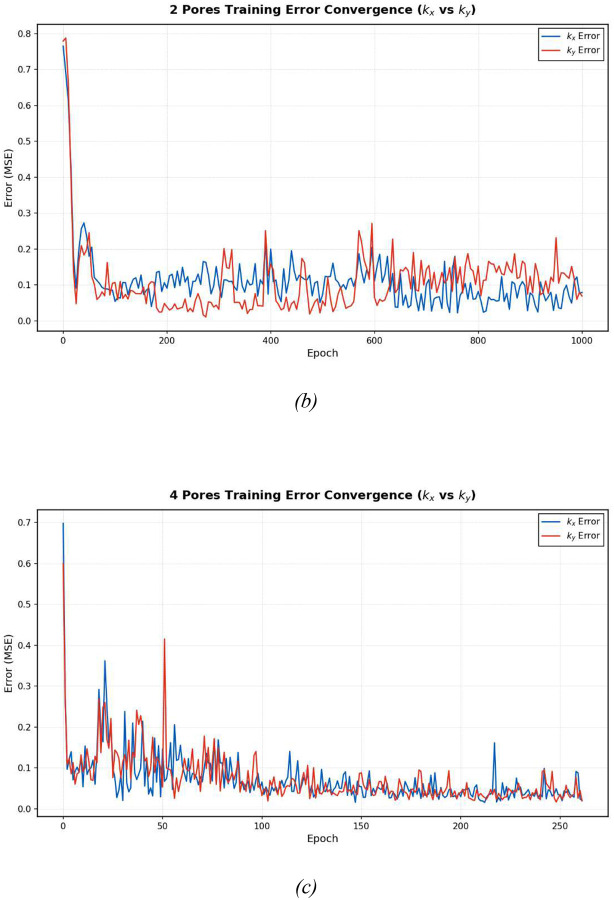

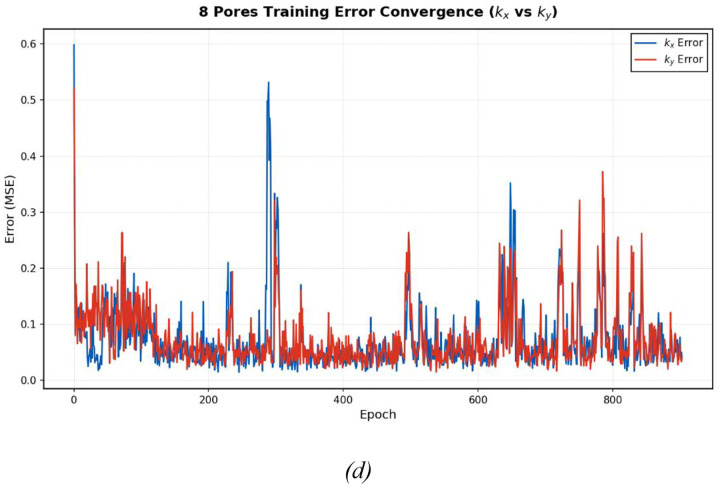
Training process for GANs trained on microstructures with varying pore counts. Each graph tracks the k_x_ and k_y_ errors over the training process The networks successfully minimize error for both directional conductivities.

The evolution of the FEM-validated conductivity prediction error during training is shown in Figure 13. The error is reported as the average relative error between the target conductivities and the FEM-computed conductivities of the generated microstructures, evaluated separately for the *x*- and *y*-directions. This metric directly reflects inverse design performance and avoids reliance on adversarial loss values, which are often difficult to interpret in GAN training.

As shown in Figure 13, all models exhibit a clear initial reduction in conductivity prediction error during the early stages of training, indicating rapid learning of the dominant microstructure–property relationships. In most cases, the error decreases substantially within the first 100–200 epochs and then gradually approaches a plateau. This behavior suggests that the GAN converges relatively quickly to a stable inverse mapping between thermal conductivities and microstructure geometry.

Superimposed on this overall convergence trend are occasional spikes in prediction error. These fluctuations are characteristic of adversarial training and arise from the competitive interaction between the generator and the discriminator. Temporary imbalances between the two networks can lead to short-lived degradation in generator performance before stability is re-established. Importantly, in all cases considered here, the model consistently recovers from these fluctuations and returns to a low-error regime, indicating robust training behavior rather than mode collapse or divergence.

The conductivity prediction errors in the *x*- and *y*-directions closely track one another throughout training, as shown in Figure 13. This symmetry reflects both the isotropic treatment of the two directions in the governing equations and the effectiveness of the conditional inputs in guiding the generator toward the correct directional thermal response. The absence of systematic divergence between *k*_*x*_ and *k*_*y*_ errors further indicates that the GAN does not preferentially favor one direction over the other during training.

Differences in convergence behavior are observed among models with different pore counts. Models trained on microstructures with fewer pores generally reach their error plateau more rapidly, which can be attributed to the lower geometric complexity of the corresponding design space. In contrast, models trained on microstructures with higher pore counts require more epochs to stabilize, reflecting the increased difficulty of accurately learning fine geometric details associated with small, closely spaced pores. Nevertheless, all models achieve stable convergence within the allotted training duration.

Overall, the training dynamics demonstrate that the proposed conditional GAN framework exhibits stable and reliable convergence across a range of microstructural complexities. The combination of rapid initial learning, recovery from adversarial instabilities, and consistent longterm performance supports the robustness of the training strategy and provides confidence in the reproducibility of the inverse design results reported in this study.

## 3. Conclusion

This work demonstrates a physics-guided generative inverse design framework for thermally anisotropic microstructures that integrates high-fidelity FEM simulations with conditional Wasserstein GANs. By grounding the learning process in physics-consistent data and validating inverse predictions through closed-loop FEM re-simulation, the proposed approach enables direct, one-shot generation of realistic and manufacturable microstructures with prescribed directional thermal conductivities.

Unlike physics-informed neural networks, which typically require iterative optimization and struggle with high-dimensional, non-unique inverse problems, the present framework performs inverse design through direct generative sampling. Compared to diffusion-based generative models, which often rely on surrogate property predictors or computationally intensive guided sampling, the proposed approach enforces physical consistency through exact numerical simulation, offering a scalable and interpretable alternative for physics-governed inverse design.

Extensive FEM validation confirms that the generated microstructures achieve the target thermal conductivities with typical relative errors below 5–10% across a wide range of anisotropy conditions. Moreover, despite the absence of explicit geometric constraints, the GAN successfully preserves pore count, ellipticity, and orientation consistency, indicating that meaningful structure–property relationships are learned directly from physics-consistent data.

Overall, this study establishes physics-guided generative modeling as a robust paradigm for inverse microstructure design, bridging high-fidelity numerical simulation and data-driven generation without reliance on surrogate physics models. While the present work focuses on computational inverse design, the framework is readily extensible to three-dimensional microstructures, multi-objective optimization, and other physics-governed structure–property relationships.

## 4. Experimental Procedure and Methods

Detailed descriptions of the finite element simulations, dataset construction, generative modeling, and evaluation procedures are provided in the Supplementary Material.

### 4.1 Physics-Based Dataset Construction and Bias Mitigation

A large, physics-consistent dataset was constructed by generating microstructures containing multiple elliptical pores and computing their effective thermal conductivities using finite element method (FEM) simulations. Each microstructure was represented as a binary image, and directional thermal conductivities along orthogonal directions were obtained from steady-state heat conduction simulations. The resulting dataset consists of paired microstructural images and effective thermal properties, forming the basis for inverse generative modeling.

Analysis of randomly generated microstructures reveals a pronounced bias toward weakly anisotropic and high-conductivity configurations, as such geometries occupy a disproportionately large region of the parameter space. This bias limits the ability of conditional generative models to accurately learn inverse mappings across the full range of anisotropy conditions. To address this issue, a geometry-aware sampling strategy was employed to promote balanced coverage of the anisotropy space while maintaining physically realistic and manufacturable microstructures.

This physics-guided dataset construction improves training stability and inverse prediction accuracy by ensuring that the generative model is exposed to a representative distribution of target properties.

### 4.2 Conditional Generative Modeling and FEM-Based Inverse Validation

A conditional generative adversarial network (GAN) was trained to learn the inverse mapping from target directional thermal conductivities to corresponding microstructural images. The model was trained exclusively on physics-consistent data generated via FEM simulations, without incorporating surrogate property predictors or explicit physics-based regularization into the learning objective.

After training, inverse generation was performed in a one-shot manner by providing target thermal conductivities as conditioning inputs to the generator. Owing to the intrinsic non-uniqueness of the inverse problem, multiple distinct microstructures may satisfy the same target properties. Accordingly, inverse performance was evaluated through physics-based validation rather than image similarity: generated microstructures were re-simulated using FEM, and the resulting effective thermal conductivities were compared with the prescribed targets.

This closed-loop validation strategy directly assesses whether the generated microstructures satisfy the governing physical behavior, providing a rigorous and application-relevant measure of inverse design accuracy.

### 4.3 Microstructure Evaluation and Inverse Design Performance

The inverse design performance of the proposed framework was evaluated using both physics-based and geometry-based metrics. Primary evaluation focused on the agreement between target thermal conductivities and those obtained from FEM re-simulation of generated microstructures, providing a direct measure of physical fidelity. Errors were quantified independently along orthogonal directions to assess anisotropy preservation.

To assess geometric realism and consistency, generated microstructures were further analyzed in terms of pore count, ellipticity, and orientation distributions. These metrics capture key geometric characteristics that influence anisotropic heat transport and reflect constraints imposed by realistic manufacturing processes. Importantly, these geometric properties were not explicitly enforced during training, allowing their preservation to serve as an indicator of the model’s ability to learn physically meaningful structure–property relationships.

Together, these evaluation metrics demonstrate that the proposed framework can generate diverse, physically consistent microstructures that satisfy prescribed anisotropic thermal properties while maintaining realistic geometric features.

## Supplementary Material

This is a list of supplementary files associated with this preprint. Click to download.

- Supplementarymaterial.docx

## Figures and Tables

**Table 1.** Representative training microstructures for different target thermal conductivities, including cases with strong x and y-direction anisotropy, and near-isotropic thermal behavior.

Number of ellipses	*k*_*x*_ ≫ *k*_*y*_	*k*_*x*_ ~ *k*_*y*_	*k*_*x*_ ≪ *k*_*y*_
1	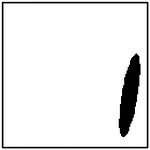 *k*_*x*_ = 0.93; *k*_*y*_ = 0.76	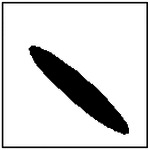 *k*_*x*_ = 0.63; *k*_*y*_ = 0.58	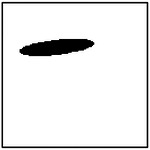 *k*_*x*_ = 0.77; *k*_*y*_ = 0.95
2	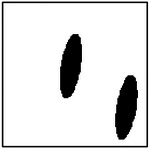 *k*_*x*_ = 0.87; *k*_*y*_ = 0.68	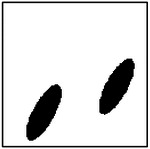 *k*_*x*_ = 0.80; *k*_*y*_ = 0.72	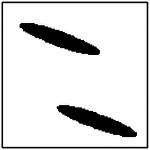 *k*_*x*_ = 0.57; *k*_*y*_ = 0.81
4	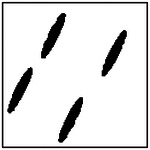 *k*_*x*_ = 0.83; *k*_*y*_ = 0.65	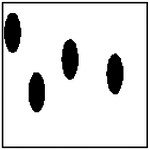 *k*_*x*_ = 0.85; *k*_*y*_ = 0.73	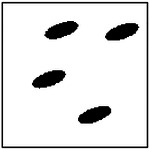 *k*_*x*_ = 0.78; *k*_*y*_ = 0.88
8	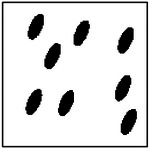 *k*_*x*_ = 0.83; *k*_*y*_ = 0.73	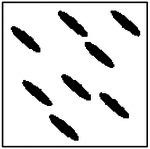 *k*_*x*_ = 0.68; *k*_*y*_ = 0.72	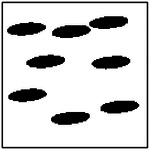 *k*_*x*_ = 0.52; *k*_*y*_ = 0.82

**Table 2.** Representative GAN-generated microstructures conditioned on different target thermal conductivities, including cases with strong x-direction anisotropy, strong y-direction anisotropy, and near-isotropic thermal behavior. All generated microstructures exhibit non-overlapping, similarly sized, and approximately aligned elliptical pores.

Number of ellipses	*k*_*x*_ ≫ *k*_*y*_	*k*_*x*_ ~ *k*_*y*_	*k*_*x*_ ≪ *k*_*y*_
1	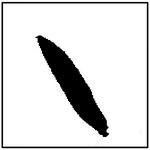 *k*_*x*_ = 0.80; *k*_*y*_ = 0.65	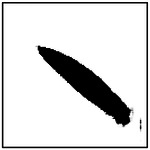 *k*_*x*_ = 0.70; *k*_*y*_ = 0.70	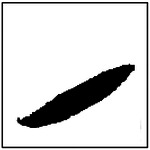 *k*_*x*_ = 0.60; *k*_*y*_ = 0.80
2	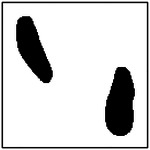 *k*_*x*_ = 0.80; *k*_*y*_ = 0.60	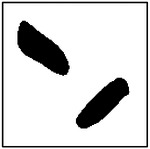 *k*_*x*_ = 0.70; *k*_*y*_ = 0.70	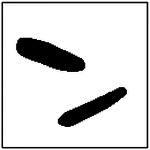 *k*_*x*_ = 0.60; *k*_*y*_ = 0.80
4	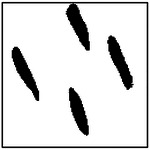 *k*_*x*_ = 0.85; *k*_*y*_ = 0.65	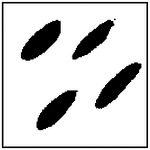 *k*_*x*_ = 0.70; *k*_*y*_ = 0.70	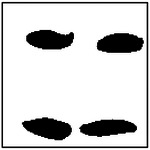 *k*_*x*_ = 0.60; *k*_*y*_ = 0.80
8	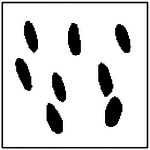 *k*_*x*_ = 0.85; *k*_*y*_ = 0.65	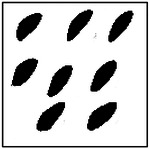 *k*_*x*_ = 0.65; *k*_*y*_ = 0.65	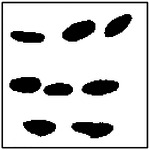 *k*_*x*_ = 0.60; *k*_*y*_ = 0.80

**Table 3.** Sample of microstructures generated from target conductivities. Their actual conductivities are calculated, and the generator errors are shown.

Input Conductivities	*k*_*x*_ = 0.70	*k*_*x*_ = 0.85	*k*_*x*_ = 0.90	*k*_*x*_ = 0.65
	*k*_*y*_ = 0.90	*k*_*y*_ = 0.90	*k*_*y*_ = 0.75	*k*_*y*_ = 0.65
Generated Microstructure	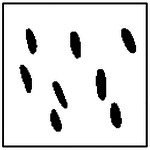	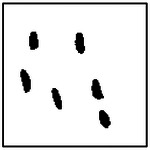	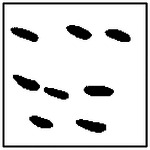	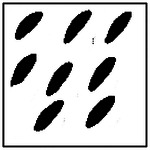
FEM-Reevaluated Conductivities	*k*_*x*_ = 0.73	*k*_*x*_ = 0.86	*k*_*x*_ = 0.87	*k*_*x*_ = 0.59
	*k*_*y*_ = 0.88	*k*_*y*_ = 0.93	*k*_*y*_ = 0.72	*k*_*y*_ = 0.68
Thermal Conductivity Error	*err*_*x*_ = 0.03	*err*_*x*_ = 0.01	*err*_*x*_ = 0.03	*err*_*x*_ = 0.06
	*err*_*y*_ = 0.02	*err*_*y*_ = 0.03	*err*_*y*_ = 0.03	*err*_*y*_ = 0.03

**Table 4.** Summary of FEM-validated thermal conductivity prediction errors for GAN-generated microstructures. Median relative errors and interquartile ranges (IQR) are reported for the predicted k_x_and k_y_across different pore counts (1, 2, 4, and 8 pores) and three anisotropy regimes: x-dominant anisotropy (k_x_ ≫ k_y_), near-isotropic behavior (k_x_ ≈ k_y_), and y-dominant anisotropy (k_y_ ≫ k_x_). For each regime, 20 randomly selected target conductivity pairs were used as inputs to the GAN.

	X-Dominant Anisotropy				Near Isotropic				Y-Dominant Anisotropy			
	*k*_*x*_ − *k*_*y*_ > 0.2				|*k*_*x*_ − *k*_*y*_| ≤ 0.2				*k*_*y*_ − *k*_*x*_ > 0.2			
# Holes	1	2	4	8	1	2	4	8	1	2	4	8
Median Error (x)	0.035	0.028	0.023	0.049	0.027	0.022	0.024	0.045	0.063	0.057	0.043	0.055
Median Error (y)	0.043	0.072	0.012	0.043	0.039	0.034	0.042	0.031	0.048	0.015	0.068	0.069
IQR (x)	0.027	0.031	0.056	0.030	0.028	0.020	0.014	0.040	0.061	0.144	0.024	0.047
IQR (y)	0.038	0.042	0.016	0.030	0.026	0.016	0.042	0.032	0.048	0.022	0.048	0.032
